# Editorial: Metal Hydride-Based Energy Storage and Conversion Materials

**DOI:** 10.3389/fchem.2020.00675

**Published:** 2020-09-04

**Authors:** Yongfeng Liu, Hai-Wen Li, Zhenguo Huang

**Affiliations:** ^1^State Key Laboratory of Silicon Materials, School of Materials Science and Engineering, Zhejiang University, Hangzhou, China; ^2^Platform of Inter/Transdisciplinary Energy Research, International Research Center for Hydrogen Energy, International Institute for Carbon-Neutral Energy Research, Kyushu University, Fukuoka, Japan; ^3^School of Civil & Environmental Engineering, University of Technology Sydney, Ultimo, NSW, Australia

**Keywords:** metal hydrides, complex hydrides, energy storage, hydrogen storage, catalysis

Energy storage and conversion materials are of critical importance in the development and utilization of new renewable clean energies (Li et al., 2016). Hydrogen, as an ideal energy carrier that can be transportable, storable, and convertible, has the potential to become a solution to energy security, resource availability, and environmental compatibility (Martin et al., 2020). Storing hydrogen in a safe, effective and economic way, however, is a great challenge in the development of a hydrogen-based economy, because of the extremely low volumetric density (0.0899 kg m^−3^) at ambient condition (Schlapbach and Züttel, 2001). Compared to pressurizing gaseous or liquefying hydrogen, storing hydrogen in metal hydride has definite advantages in terms of gravimetric and volumetric density, safety, and energy efficiency, for both mobile and stationary applications (Wu, 2008; He et al., 2019; Ouyang et al., 2020). Criteria developed by the US Department of Energy (DOE) for onboard hydrogen storage for light-duty fuel cell vehicles include 6.5 wt% of systematic gravimetric density and 50 kg H_2_ m^−3^ of volumetric density along with other stringent properties such as operating temperature (<85°C), extended cycle-life, fast kinetics, safety, and cost. Therefore, in the last decade tremendous efforts have been devoted to the research and development of light metal hydrides, including MgH_2_, alanates, borohydrides, amides/imides, which hold sufficiently high hydrogen capacity (Orimo et al., 2007; Hansen et al., 2016; Yu et al., 2017; Liu et al., 2018; Schneemann et al., 2018; Zhou et al., 2019; Hirscher et al., 2020).

This special issue of *Metal Hydride-Based Energy Storage and Conversion Materials* is focused on the synthesis, catalyst development, and nano-structuring of light metal hydrides (MgH_2_, AlH_3_, NaAlH_4_, and LiBH_4_) as hydrogen storage media. The eight contributions to this special issue highlight that metal hydrides are promising candidates for high density hydrogen storage.

Catalysts prove effective in reducing the reaction energy barriers for hydrogen absorption and desorption in Mg-based materials. Ding et al. report the catalytic activity of Co-Ni nanocatalyst with different compositions and morphology for hydrogen storage reaction of MgH_2_. The partial replacement of Ni by Co induced a change in the morphology from spherical to plate-like, which is found to be less effective toward catalytic activity, presumably due to reduced surface contact. Zeng et al. prepared Ni and TiO_2_ co-anchored on reduced graphene oxide [(Ni-TiO_2_)@rGO], which showed superior catalytic effects on the hydrogen desorption, as evidenced by the release of 1.47 wt% H_2_ by MgH_2_ within 120 min at 225°C. Wang and Deng ameliorated the performance of MgH_2_ by using a core-shell Co@N-rich carbon (CoNC) based catalyst. In their work, the MgH_2_-5 wt% CoNCo composites released up to 6.58 wt% of H_2_ in 5 min at 325°C. Liu Y. et al. present that Pd-decorated Mg nanoparticles, ranging from 40 to 70 nm, started releasing H_2_ at 216.8°C and absorbed 3.0 wt% hydrogen in 2 h at 50°C. In addition, Wu et al. investigated the effects of CeH_2.73_/CeO_2_ composites on the desorption properties of Mg_2_NiH_4_. The onset dehydrogenation temperature and activation energy of Mg_2_NiH_4_ were largely reduced when CeH_2.73_/CeO_2_ composite with the same molar ratio of hydride and oxide were used as a catalyst.

Zhang et al. reported highly stable catalytic activity of amorphous-carbon-supported TiB_2_ nanoparticles with sizes of 2–4 nm (nano-TiB_2_@C) for hydrogen storage in NaAlH_4_. They observed 5.04 wt% of hydrogen released at 140°C within 60 min, and complete hydrogenation at 100°C within 25 min under a hydrogen pressure of 100 bar. The stable catalytic function was closely related to the *in-situ* formed Ti–Al alloy, which facilitated the dissociation and formation of H–H and Al–H bonds, respectively. Liu H. et al. reported wet chemical synthesis of non-solvated rod-like α'-AlH_3_, which releases 7.7 wt% H_2_ at 120–200°C. In addition, Fan et al. synthesized a flexible, water-resistant, and air-stable hydrogen storage material (PMMA-LiBH_4_/GMF), which consists of LiBH_4_ nanoparticles confined by poly (methylmethacrylate) (PMMA) and reduced graphene oxide (rGO) modified melamine foam (GMF). Interestingly, the onset dehydrogenation temperature of PMMA-LiBH_4_/GMF was reduced to 94°C and desorbed 2.9 wt% hydrogen within 25 min at 250°C. This simple preparation process sheds light on how to improve the performance of LiBH_4_-based hydrogen storage materials.

As the Guest Editors of this topic issue, we would like to thank all the authors for their contributions and all the referees for their thoughtful suggestions and insights. We hope this special issue will inspire research and interest in light metal hydrides for hydrogen storage, and that future endeavors will contribute to the realization of a hydrogen economy that is sustainable and environmentally friendly.

## Author Contributions

All authors listed have made a substantial, direct and intellectual contribution to the work, and approved it for publication.

## Conflict of Interest

The authors declare that the research was conducted in the absence of any commercial or financial relationships that could be construed as a potential conflict of interest.
